# Whose Decision Is It? Perspectives on Agency Involvement in Determining Paid Caregivers' Roles in Dementia Care

**DOI:** 10.1093/geroni/igab046.836

**Published:** 2021-12-17

**Authors:** Jennifer Reckrey, Deborah Watman, Emma Tsui

**Affiliations:** 1 Icahn School of Medicine at Mount Sinai, Icahn School of Medicine at Mount Sinai, New York, United States; 2 Icahn School of Medicine at Mount Sinai, New York, New York, United States; 3 CUNY Graduate School of Public Health & Health Policy, CUNY Graduate School of Public Health & Health Policy, New York, United States

## Abstract

Individuals living at home with dementia often rely on a team of caregivers and health care providers. Yet little is known about how the role of paid caregivers within this team is determined. We identified patients with moderate to severe dementia (n=9) and conducted individual interviews with their care teams (family caregiver, paid caregiver, physician) (n=27) to explore perspectives on paid caregiver roles. Participants disagreed on who determined the paid caregiver’s role. Agencies were perceived to set limitations on the scope of care (particularly by physicians) but agency care plans were often seen as inadequate and failing to capture important nuances of care. Most family caregivers believed they should guide what paid caregivers did in the home, while most paid caregivers reported relying on their own experience and knowledge. Understanding and addressing these differing perceptions is critical to improving the quality of paid care in the home.

